# 

**Published:** 1957-07

**Authors:** 


					'E3?<?
THE
MEDICAL JOURNAL
OF THE
SOUTH-WEST
Incorporating
The Bristol Medico-Chirurgical Journal
M)G 16 ?57
,
f- . i >
Ju77 2957
vol.
?2 (iii). No. 26^ Price 4/-

				

